# Alternative polyadenylation of ZEB1 promotes its translation during genotoxic stress in pancreatic cancer cells

**DOI:** 10.1038/cddis.2017.562

**Published:** 2017-11-09

**Authors:** Ilaria Passacantilli, Valentina Panzeri, Pamela Bielli, Donatella Farini, Emanuela Pilozzi, Gianfranco Delle Fave, Gabriele Capurso, Claudio Sette

**Affiliations:** 1 Department of Biomedicine and Prevention, Section of Anatomy, University of Rome ‘Tor Vergata’, Rome, Italy; 2 Department of science medical/chirurgic and translational medicine, University of Rome ‘Sapienza’, Rome, Italy; 3 Laboratory of Neuroembryology, Fondazione Santa Lucia IRCCS, Rome, Italy

## Abstract

Pancreatic ductal adenocarcinoma (PDAC) is characterized by extremely poor prognosis. The standard chemotherapeutic drug, gemcitabine, does not offer significant improvements for PDAC management due to the rapid acquisition of drug resistance by patients. Recent evidence indicates that epithelial-to-mesenchymal transition (EMT) of PDAC cells is strictly associated to early metastasization and resistance to chemotherapy. However, it is not exactly clear how EMT is related to drug resistance or how chemotherapy influences EMT. Herein, we found that ZEB1 is the only EMT-related transcription factor that clearly segregates mesenchymal and epithelial PDAC cell lines. Gemcitabine treatment caused upregulation of ZEB1 protein through post-transcriptional mechanisms in mesenchymal PDAC cells within a context of global inhibition of protein synthesis. The increase in ZEB1 protein correlates with alternative polyadenylation of the transcript, leading to shortening of the 3' untranslated region (UTR) and deletion of binding sites for repressive microRNAs. Polysome profiling indicated that shorter ZEB1 transcripts are specifically retained on the polysomes of PDAC cells during genotoxic stress, while most mRNAs, including longer ZEB1 transcripts, are depleted. Thus, our findings uncover a novel layer of ZEB1 regulation through 3'-end shortening of its transcript and selective association with polysomes under genotoxic stress, strongly suggesting that PDAC cells rely on upregulation of ZEB1 protein expression to withstand hostile environments.

Pancreatic ductal adenocarcinoma (PDAC) is among the deadliest human cancers, with rate of overall survival at 5 years from diagnosis being less than 5%.^1^ Late diagnosis and the highly metastatic behavior of PDAC cells substantially contribute to such poor prognosis.^2^ Moreover, despite improvement in surgical techniques and introduction of novel chemotherapeutic approaches, PDAC patients rapidly develop resistance to therapies and progress to advanced, incurable stages.

Epithelial-to-mesenchymal transition (EMT), namely the ability of an epithelial cell to acquire a fibroblast-like shape, is among the biological processes promoting metastatic dissemination of epithelial cancers, including PDAC.^3, 4^ EMT is a physiological process that underlies cell migration and organ colonization in the developing embryo. However, it can be re-activated upon neoplastic transformation of epithelial cells, permitting them to reach distal tissues and to give rise to metastasis.^5, 6^ In PDAC mouse models, EMT allows dissemination of cancer cells even before the primary tumor is fully visualized.^7^ Furthermore, acquisition of the mesenchymal phenotype seems to be related to drug resistance of PDAC cells,^8, 9, 10, 11^ in particular to gemcitabine, the standard first-line treatment for PDAC. However, the specific molecular mechanisms by which the mesenchymal phenotype contributes to chemoresistance are not fully understood.

EMT is driven by global gene expression re-programming operated by transcription factors (TFs), such as ZEB1, ZEB2, SNAIL, SLUG and TWIST.^12^ All these TFs activate transcription of mesenchymal genes while repressing epithelial genes. However, emerging evidence strongly suggests that not all EMT programs are equal *in vivo*. Indeed, studies using PDAC mouse models clearly showed that EMT triggered by ZEB1 promotes cell plasticity and metastasis,^13^whereas SNAIL and TWIST had no effect on invasion or metastasis while they favored resistance to chemotherapy.^11^ Furthermore, ZEB1 was shown to play a key role in early dissemination of PDAC cells.^7^ Thus, regulation of ZEB1 expression is highly relevant for PDAC onset and progression.

Genotoxic stress elicited by most chemotherapeutic drugs often turns on pro-survival pathways that result in selection of resistant cells. Modulation of post-transcriptional pre-mRNA processing is a key step in the fine-tuned regulation of gene expression programs during both EMT and acquisition of drug resistance.^6, 14^ In the present work, we found that gemcitabine induces an increase in ZEB1 protein expression in mesenchymal PDAC cell lines, which occurred concomitantly with global inhibition of protein synthesis. Cell fractionation experiments showed that while most mRNAs are released from the polysomes following the translational block, ZEB1 mRNA remains associated with them. Mechanistically, gemcitabine treatment promoted alternative polyadenylation of ZEB1 mRNA, leading to shortening of its 3' untranslated region (UTR). This shorter ZEB1 transcript variant was more efficiently associated with polysomes than longer ZEB1 transcripts in cells exposed to the drug. Our findings highlight a novel mechanism involved in ZEB1 regulation in response to genotoxic stress and suggest that its enhanced expression offers an opportunity to PDAC cells to survive to the insult while priming them for metastatic spread.

## Results

### Gemcitabine treatment enhances ZEB1 expression in mesenchymal PDAC cell lines

Mesenchymal phenotype has been associated with resistance to chemotherapy in PDAC.^8, 9, 10, 11^ Accordingly, colony formation assays indicated thatPt45P1 and MiaPaCa-2 PDAC cells, which express the mesenchymal marker Vimentin (Supplementary Figures S1A,B), were significantly more resistant to low dosage (0.01 *μ*M) of gemcitabine than the HPDE immortalized epithelial ductal cells and HPAF-II PDAC cells (Supplementary Figure S1C), which both display an epithelial phenotype and express E-cadherin (Supplementary Figures S1A,B). At increasing doses (0.03–0.1 *μ*M), Pt45P1 cells showed higher sensitivity than MiaPaCa-2 (Supplementary Figure S1C). Likewise, cleavage of full length PARP-1 also showed higher sensitivity to apoptosis of epithelial HPAF-II cells with respect toPt45P1 and MiaPaCa-2cells (Supplementary Figure S1D), confirming the correlation between drug resistance and the mesenchymal phenotype in PDAC cell lines.

**Figure 1:**
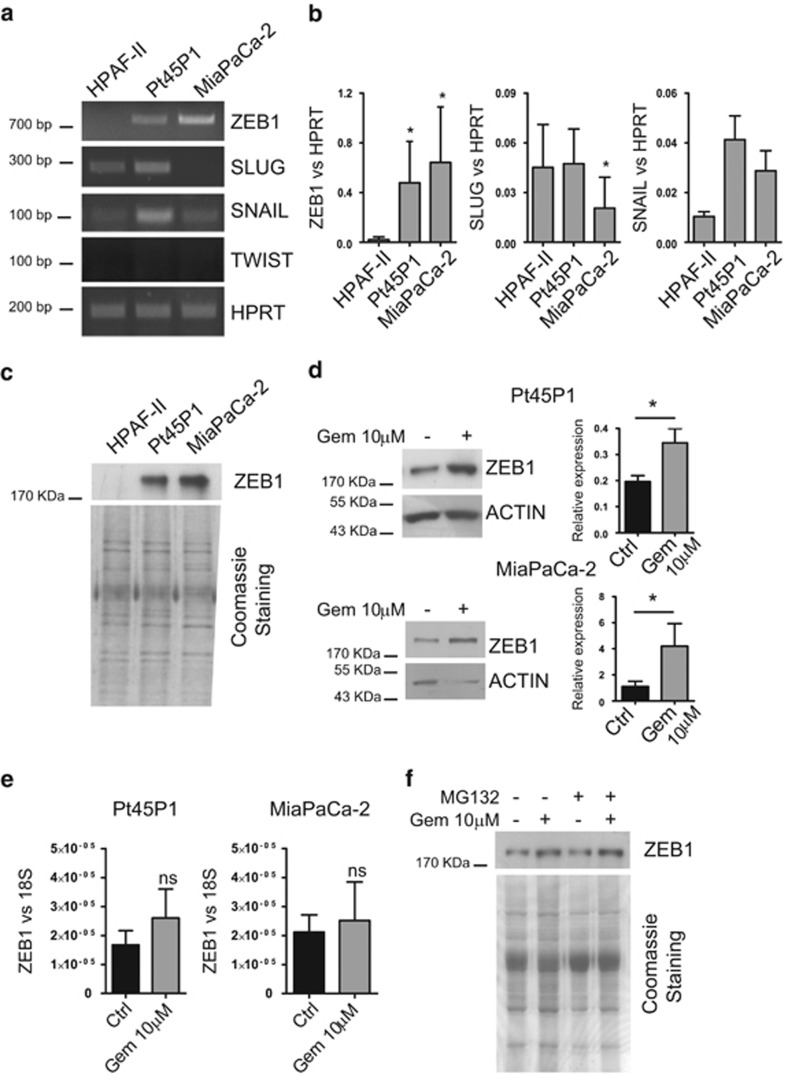
Gemcitabine induces upregulation of ZEB1 protein in mesenchymal PDAC cells. Conventional (**a**) and quantitative (**b**) RT-PCR analysis of ZEB1, SLUG, SNAIL and TWIST mRNA expression in the indicated PDAC cell lines. *HPRT* was used as housekeeping gene for normalization. (**c**) Western blot analysis of ZEB1 protein expression in the indicated PDAC cell lines. Coomassie blue staining was performed as sample loading control. (**d**) Western blot and (**e**) quantitative RT-PCR of ZEB1 expression in Pt45P1 and MiaPaCa-2 cells treated or not with gemcitabine (10 *μ*M) for 48 h. (**f**) Western blot analysis of ZEB1 protein expression in MiaPaCa-2 cells treated or not with gemcitabine (10 *μ*M) for 48 h. MG132 was added to cells in the last 8 h of treatment. Bar graphs in (**b**), (**d**) and (**e**) represent the mean±S.D. of three experiments. * *P*<0.05; n.s.=not significant

To investigate which EMT-related TF was differentially expressed in PDAC cell lines, we performed conventional (cPCR) and quantitative real-time PCR (qPCR) analyses. Notably, only ZEB1 clearly segregated the mesenchymal Pt45P1 and MiaPaCa-2 from the epithelial HPAF-II cells, whereas SLUG and SNAIL were expressed at variable levels regardless of the phenotype and TWIST levels were not detectable (Figures 1a and b). Differential ZEB1 expression between epithelial and mesenchymal PDAC cells was also observed at the protein level (Figure 1c), confirming the correlation with the gemcitabine-resistant phenotype of these cells.

Poor prognosis in PDAC is mainly due to unresponsiveness of patients to chemotherapy with gemcitabine, either as single agent or in combination with other drugs.^15^ Given the role of ZEB1 in PDAC cells drug resistance,^9, 13^ we asked whether genotoxic stress associated with chemotherapy affected its expression. Treatment with gemcitabine for 48 h elicited a significant upregulation of ZEB1 protein expression in both Pt45P1 and MiaPaCa-2 cells (Figure 1d). Notably, such increase was sustained over at least 72 h (Supplementary Figure S1E) and not accompanied by significant upregulation of ZEB1 mRNA (Figure 1e), nor by stabilization of the pre-existing ZEB1 protein, as tested by incubation of cells with the proteasome inhibitor MG132 (Figure 1f). These results suggest that gemcitabine induces upregulation of ZEB1 expression by post-transcriptional mechanism(s).

### Gemcitabine induces a global inhibition of protein synthesis in PDAC cell lines

**Figure 2:**
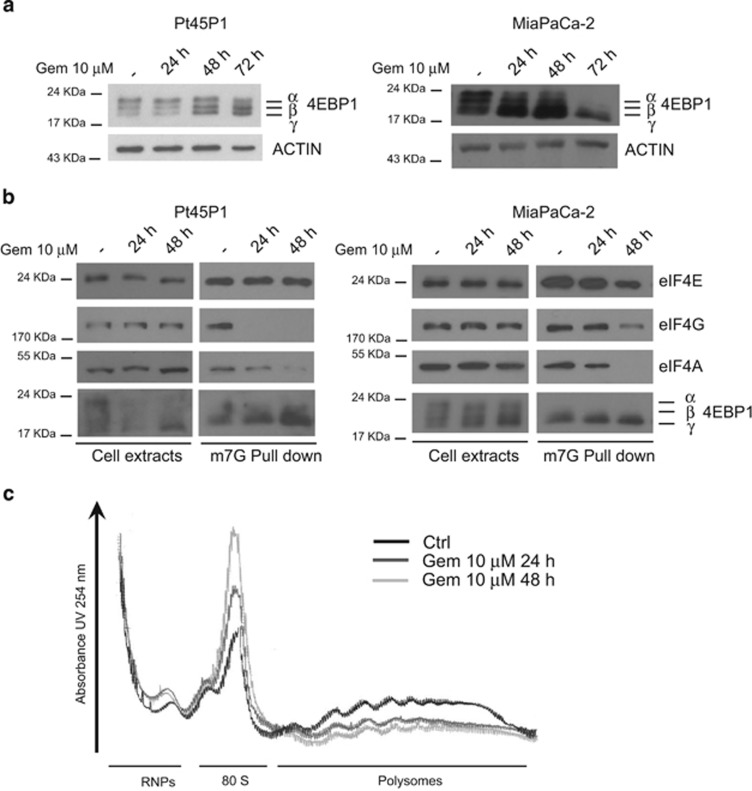
Gemcitabine causes global inhibition of cap-dependent translation in PDAC cells.(**a**) Western blot analysis of 4EBP1 in the indicated PDAC cell lines treated for increasing time with 10 *μ*M gemcitabine. Hyper- (*α*,*β*), and hypophosphorylated (*γ*) 4EBP1 are indicated. (**b**) Western blot analyses of cell extracts (left panels) and methyl-7-cap pulldown assays (right panels) using the indicated PDAC cells treated or not with 10 *μ*M gemcitabine for increasing time. Components of the eIF4F complex (eIF4E, eIF4G and eIF4A) and 4EBP1 are indicated. (**c**) Plot of the absorbance profile of fractions obtained on sucrose gradients to isolate polysomes from monosome (80S) and free RNPs in MiaPaCa-2 cells treated or not with 10 *μ*M gemcitabine for the indicated time

Acute chemotherapeutic treatments often activate responses that facilitate survival of cancer cells to genotoxic stress.^16, 17, 18, 19^ To investigate the post-transcriptional mechanism(s) underlying the increase in ZEB1 protein levels, we first monitored the impact of gemcitabine treatment on the mTORC1 pathway, a regulatory route of protein synthesis with strong relevance for human cancers.^20^ A key target of the mTORC1 kinase is the inhibitory protein 4EBP1, which binds the translation initiation factor eIF4E and prevents its assembly with eIF4G and the RNA helicase eIF4A to form the translation initiation complex eIF4F.^20^ Phosphorylation of 4EBP1 by mTORC1 causes its release from eIF4E and stimulates eIF4F assembly and cap-dependent translation. Treatment with gemcitabine caused progressive inhibition of 4EBP1 phosphorylation in Pt45P1 and MiaPaCa-2cells by 24–72 h, with increasing prevalence of the non-phosphorylated form (*γ*) of 4EBP1 (Figure 2a). To confirm the effect of genotoxic stress on the inhibition of cap-dependent translation, we performed methyl-cap pulldown assays to isolate the eIF4F complex.^21^ Drug treatment impaired association of eIF4A and eIF4Gwith eIF4E on methyl-cap in both cell lines, whereas binding of hypo-phosphorylated 4EBP1 was increased (Figure 2b). In line with this effect, gemcitabine caused a global decrease of mRNA loaded on polysome fractions while it increased the peaks associated with monosomes (80S) and free ribonucleoproteinparticles (RNPs), which are not engaged in translation (Figure 2c). Thus, genotoxic stress triggered by gemcitabine inhibits cap-dependent translation and generally stalls protein synthesis in PDAC cells.

### ZEB1mRNAis selectively maintained on polysomes during genotoxic stress

**Figure 3:**
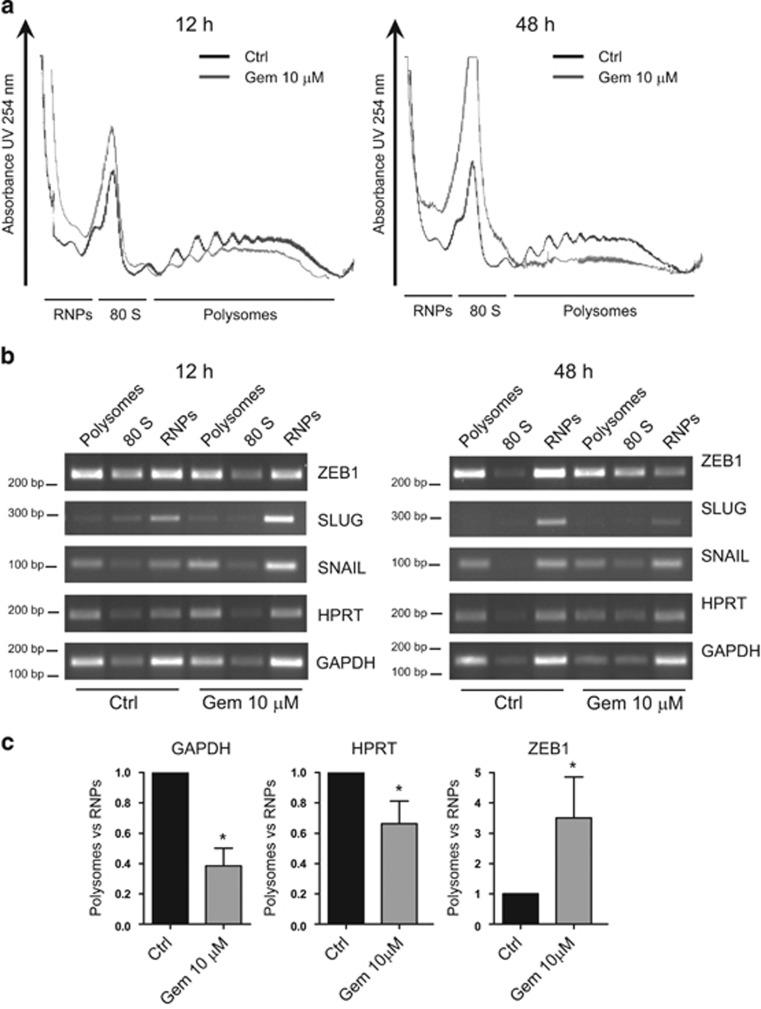
ZEB1mRNAis selectively maintained on polysomes during genotoxic stress. (**a**) Absorbance profiles of fractions obtained on sucrose gradients to isolate polysomes from monosome (80S) and free RNPs in MiaPaCa-2 cells treated or not with 10 *μ*M gemcitabine for 12 h (left panel) or 48 h (right panel). (**b**) Conventional RT-PCR analysis of the distribution of the indicated mRNAs in polysome, 80S and RNP fractions from cells shown in (**a**).(**c**) Quantitative RT-PCR of the ratio of distribution between polysome and RNP fractions of the indicated mRNA. Data are normalized for the ratio in control cells and represent the mean±S.D. of three experiments. * *P*<0.05

Since upregulation of ZEB1 in PDAC cells occurred in a context of global protein synthesis inhibition, we specifically analyzed the profile of ZEB1 mRNA distribution in polysome fractionation experiments.ZEB1 transcript, like those of the housekeeping genes HPRT and GAPDH, was almost equally distributed between polysome and RNP fractions in control cells, and this pattern was not substantially affected by 12 h treatment with gemcitabine (Figures 3a and b). However, while prolonged exposure to the drug (48 h) caused a drastic decrease in polysomal loading of the HPRT and GAPDH mRNAs, ZEB1 mRNA was actively retained on polysomes with respect to the free RNP fraction (Figures 3a and b). Notably, this regulation was specific for ZEB1, as transcripts of other EMT-related TFs (SLUG and SNAIL) behaved like HPRT and GAPDH mRNAs and in line with the global decrease of mRNA translation (Figures 3a and b). Quantitative analyses by qPCR confirmed that treatment with gemcitabine significantly increased the polysome/RNP ratio for ZEB1 mRNA (Figure 3c) while decreasing that of HPRT and GAPDH mRNAs.

Polysomal recruitment of mRNAs upon inhibition of cap-dependent translation can be mediated by internal ribosome entry segments (IRES)-mediated translation.^22^ IRES are structured sequences located in the 5′UTR of mRNAs and promote ribosome recruitment. Search in the Ensemble database (http://www.ensembl.org/index.html) revealed annotation of two classes of 5′ UTR for ZEB1 transcripts encoded by alternative first exons. Exon 1-containing transcripts encode for short 5′ UTRs with unstructured nucleotide sequence (ENST00000320985.14, 100 bp; ENST00000560721.6, 22 bp; ENST00000542815.7, 71 bp; ENST00000361642.9, 63 bp), whereas exon 1C encodes a longer, structured 5′ UTR of 392 nucleotides (ENST00000446923.6) that resembles an IRES sequence (Supplementary Figure S2A). To test whether this sequence acted as IRES, we inserted it into the intercistronic region of the pRF construct, which contains the Renilla and Firefly luciferase genes as upstream and downstream cistrons, respectively (Supplementary Figure S2B).^23^ Transfection of this construct into MiaPaCa-2 cells indicated that, unlike the Myc IRES element, the exon 1C-encoded sequence was unable to induce expression of the downstream cistron regardless of gemcitabine treatment (Supplementary Figure S2B), suggesting that it does not possess IRES activity. Accordingly, we found that ZEB1 transcripts containing this longer 5′ UTR were not selectively retained on polysomes upon treatment with gemcitabine (Supplementary Figure S2C). Thus, alternative 5′ UTR usage and/or IRES-dependent translation are unlikely responsible for the increased translation of ZEB1 upon gemcitabine-induced stress.

### Alternative polyadenylation of ZEB1 supports its translation upon genotoxic stress

**Figure 4:**
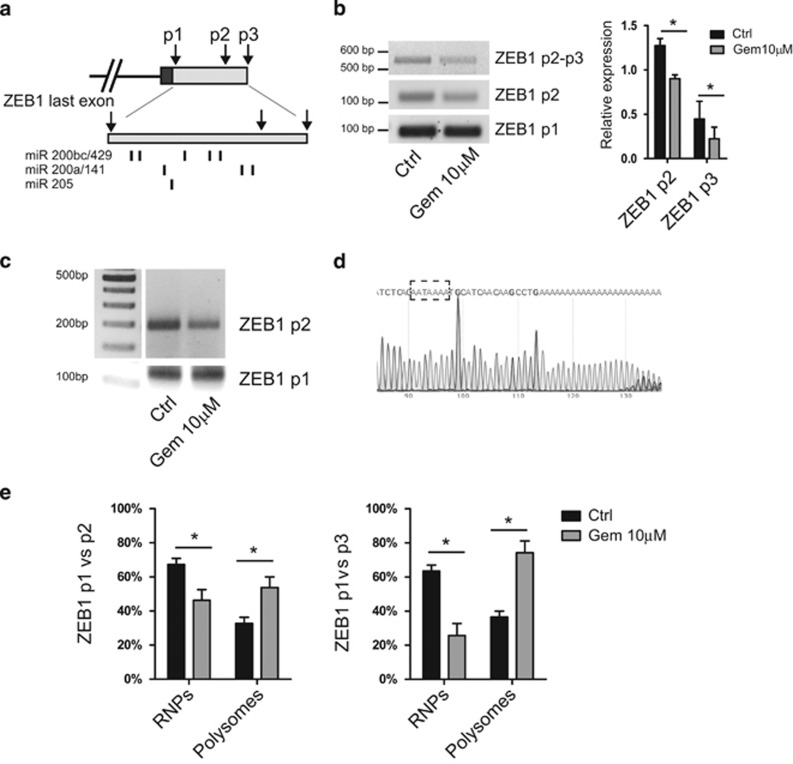
ZEB1 mRNA 3′-end shortening supports its translation upon genotoxic stress. (**a**) Schematic representation of the 3′ UTR region of ZEB1. Arrows indicate position of the three PASs present in the last exon of ZEB1. Inset shows magnification of the 3′ UTR region with the position of the binding sites for microRNAs known to exert repression of ZEB1 expression in MiaPaCa-2 cells located between p1 and p2 (arrows). (**b**) RT-PCR analyses of the expression of the last exon-encoded portion of the ZEB1 mRNA in cells treated or not with 10 *μ*M gemcitabine for 48 h. Primers p2–p3 were used to amplify transcripts terminating at p3, primers p2 to amplify transcripts terminating at p2 or p3, primers p1 for all transcripts. Bar graph shows quantitative densitometric analyses (mean±S.D. of three experiments; *P<0.05). (**c**) 3′-end RACE analyses of cells described in (**b**) using forward primers located upstream of p2 or p1, as indicated. (**d**) Sequence analysis of the band amplified by 3′-end RACE using the p2 primer confirming the PAS and polyadenylation of the transcript. (**e**) Quantitative RT-PCR of the ratio of distribution between RNP and polysome fractions of mRNAs. Data represent the ratio between p1 and p2 or p3 amplified signals to highlight shortening of the transcript and its relative distribution between polysomes and RNPs (mean±S.D. of three experiments; * *P*<0.05)

Translation regulation can be also modulated by changes in the 3′ UTR of target mRNAs, which result from alternative polyadenylation (APA) regulation.^24^ Three main alternative polyadenylation signals (PAS) are present in the ZEB1 last exon (indicated as p1–p3 from proximal to distal in Figure 4a). Notably, binding sites for microRNAs that repress ZEB1 expression by targeting its 3′UTR (miR-200 family and miR-205)^9, 25^ are located between the p1 and p2 PAS (Figure 4a).By using reverse primers located upstream of the p1, p2 or p3 PAS, we observed that treatment of MiaPaCa-2 cells with gemcitabine caused shortening of the 3'UTR of ZEB1, with selective decrease of transcripts terminating at the p2 and p3 PAS (Figure 4b). To verify that shortening was due to APA regulation, we performed 3'-RACE experiments in MiaPaCa-2 cells treated or not with gemcitabine. Drug treatment caused reduced termination of the ZEB1 transcript at the p2 PAS, without affecting termination at the p1 PAS (Figure 4c). Sequencing of the PCR product verified the presence of a PAS at the p2 region and polyadenylation (Figure 4d), thus confirming that ZEB1 APA regulation is sensitive to stress.

Next, we asked whether APA resulted in differential polysomal loading of ZEB1 alternative transcripts. Polysome fractionation experiments in control and gemcitabine-treated samples revealed that the p1/p2 ratio and p1/p3 ratio were significantly increased in the polysome fraction and reduced in the RNP fraction obtained from MiaPaCa-2 cells treated with gemcitabine (Figure 4e). These experiments indicate that gemcitabine causes a general shortening of the 3'UTR of ZEB1, and that transcripts containing the shorter 3'UTR are more efficiently retained on polysomes than longer transcripts during genotoxic stress in PDAC cells.

## Discussion

Cancer cells display remarkable adaptability to adverse environments, which likely contributes to acquisition of resistance to therapeutic treatments.^18^ This is particularly true for PDAC cells and manifests in the failure of efficacious therapies for patients after years of efforts and research in the field.^2, 15^ One feature that is related to the refractoriness of PDAC cells to treatments is their plasticity. Indeed, acquisition of a mesenchymal phenotype by epithelial PDAC cells is thought to be responsible for both metastatic behavior and resistance to chemotherapy.^7, 8, 9, 10, 11, 12, 13^ Although EMT can be set in motion by several TFs,^12^ ZEB1 plays a unique and prominent role in PDAC metastasization and resistance to treatments.^13^ Nevertheless, how and when ZEB1 expression is altered in response to chemotherapy is still largely unknown. Herein, we found that upregulation of ZEB1 expression is an early event in such response, which occurs within 24–48 h from exposure of PDAC cells to gemcitabine. Importantly, ZEB1 upregulation is driven by a post-transcriptional mechanism that occurs in the absence of changes in ZEB1 transcript levels and in a general context of translational inhibition. In this scenario, while most mRNAs are depleted from the polysomes, ZEB1 transcripts remain associated with the translational machinery and likely insure continuous translation of this key TF under gemcitabine-elicited genotoxic stress. Thus, our findings suggest that the peculiar post-transcriptional regulation of ZEB1 expression represents a feedback mechanism set in motion by mesenchymal PDAC cells to withstand adverse conditions caused by chemotherapy.

Protein synthesis is an energy-consuming process and cells tune it down under various types of stress, including genotoxic stress elicited by most chemotherapeutic drugs.^22^Nevertheless, some proteins are instrumental to survive under stress conditions and need to be translated. In most cases, regulation of translation under stress is conferred by the 5' and 3'UTR of target transcripts. In our study, we provide evidence that changes in the 3'UTR of ZEB1 contribute to its sustained expression upon genotoxic stress. It is well established that many microRNAs elicit translational repression by binding to complementary sequences in the 3'UTR of target transcripts. Recent evidence suggests that most human 3'UTR sequences contain more than one PAS and that alternative PAS selection leads to expression of multiple transcript variants through APA.^24^ In general, progressive shortening of the 3'UTR through selection of proximal PASs should relieve repression by microRNAs and promote translation. Accordingly, it has been observed that global 3'UTR shortening correlates with high proliferative rates whereas lengthening occurs during cell differentiation.^24^The 3'UTR of ZEB1 is particularly long (~2500 nucleotides) and contains putative binding sites for many microRNAs, including members of the miR-200 family that repress its expression in cancer epithelial cells.^25^ Genotoxic stress caused shortening of the 3'UTR, with selective depletion of transcripts terminating at the distal PASs (p2 and p3) in cells treated with gemcitabine. Notably, most of the microRNA binding sites, including all the sites for the repressive miR-200 members, map between p1 and p2. APA leading to shortening of the 3'UTR should relieve translational repression of ZEB1 by these microRNAs. In line with this hypothesis, treatment with gemcitabine differentially affected the association of ZEB1 transcript variants with polysomes, with proportional increase in those terminating at p1 and corresponding depletion of transcripts terminating at p2 and p3. Thus, progressive shortening of the ZEB1 transcript may promote its translation by removing inhibition by microRNAs and favoring its recruitment onto polysomes. Since ZEB1 and miR-200 members are under mutual control in a negative feedback loop,^9^we suggest that this mechanism may rapidly shift the balance in favor of ZEB1 and mesenchymal features in PDAC cells exposed to gemcitabine.

APA is regulated by changes in the activity and/or expression of key components of the cleavage and polyadenylation complex, which often cooperate with accessory RNA binding proteins (RBPs) that bind elements located near the regulated PAS.^24^ Some of these RBPs, like Sam68 and hnRNPH, are often upregulated in human cancers and can suppress or enhance recognition of cryptic PASs depending on the context.^26, 27^Notably, localization and function of Sam68,^28^ as well as many other RBPs,^18, 29^ is regulated upon genotoxic stress. Thus, it is possible that gemcitabine-induced stress promotes the recruitment of specific RBPs to the ZEB1 transcript, thereby affecting the choice of the proximal PAS and enhancing translational efficiency. Alternatively, APA is also modulated by the rate of transcription.^24^ Changes in the phosphorylation status of the RNA polymerase II (RNAPII) affect its elongation rate within the transcription unit and its pausing at PAS. This modulation of RNAPII dynamics can alter the time window for PAS recognition and usage.^24^ More recently, it has also been shown that reduced expression of RNAPII upon several stresses, including DNA damage, switches APA regulation from the preferred PAS to alternative ones in selected transcripts.^30^ In this regard, gemcitabine is known to reduce the transcriptional activity of PDAC cells^16^ and might contribute to the switch in ZEB1 APA by concomitantly regulating RNAPII expression or function and depleting endogenous nucleotides due to interference with their biosynthesis. These observations suggest that further mechanistic studies will be required to dissect the exact mechanism(s) involved in ZEB1 APA regulation during genotoxic stress. Furthermore, since ZEB1-driven EMT is particularly efficient to confer the highly aggressive and resistant phenotype typifying PDAC cells,^13^ our findings also suggest that development of tools to alter APA in ZEB1 may help sensitizing PDAC cells to genotoxic stress.

## Materials and methods

### Cell culture, treatments and tranfections

HPDE, HPAF-II and Pt45P1 were cultured in RPMI 1640 medium (Lonza, Switzerland) supplemented with 10% fetal bovine serum (Gibco Thermo Fisher, Waltham, MA, USA), MiaPaCa-2 cells were cultured in DMEM medium (Lonza) supplemented with 10% fetal bovine serum (Gibco). Cells were grown in a 37 °C humidified atmosphere of 5% CO_2_. Gemcitabine (Eli Lilly & Company, Indianapolis, IN, USA) was dissolved in water and stored at −20 °C.

### RT-PCR and qPCR analysis

Total RNA was extracted from cells using Trizol reagent (Invitrogen Thermo Fisher, Waltham, MA, USA) according to the manufacturer’s instructions. After digestion with RNase free DNase (Ambion Thermo Fisher, Waltham, MA, USA), RNA was resuspended in RNase free water (Sigma-Aldrich, St. Louis, MO, USA); 1 *μ*g of total RNA was retrotranscribed using M-MLV reverse transcriptase (Promega, Madison, WI, USA). Five percent of the reaction was used as template for RT-PCR analysis (GoTaq, Promega) or qPCR analysis (SYBR Green method, Roche, Germany). Primers used are listed in Supplementary Table S1.

### Protein extraction and western blot analysis

Cells were resuspended in lysis buffer (100 mM NaCl, 15 mM MgCl_2_, 2 mM EDTA, 20 mM Hepes, 10% Glycerol, 1 mM dithiothreitol, 2 mM Na-ortovanadate, Protease-Inhibitor Cocktail (Sigma-Aldrich) and 1% Triton X-100). After10 min of incubation in ice, extracts were centrifuged for 10 min at 12 000 rpm at 4 °C, supernatants were resuspended in SDS-page sample buffer and boiled for 5 min. Western blot analysis was performed as previously described.^31^ The following primary antibodies (overnight at4 °C) were used: rabbit anti-Actin (1 : 1000, Sigma-Aldrich), mouse anti-GAPDH (1 : 1000, Santa Cruz Biotechnology, Santa Cruz, CA, USA), rabbit anti-E-cadherin (1 : 1000, Santa Cruz Biotechnology), mouse anti-Vimentin (1 : 1000, Santa Cruz Biotechnology), rabbit anti-ZEB1 (1 : 1000, Sigma-Aldrich), rabbit anti-eiF4E (1 : 1000, Cell Signalling Technology, Danvers, MA, USA), rabbit 4EBP1 (1 : 1000, Cell Signalling Technology), rabbit anti PARP-1 (1 : 1000, Cell Signalling Technology), rabbit anti eiF4A (1 : 1000, Abcam, UK), rabbit anti-eIF4G (1 : 1000, Cell Signalling Technology). Secondary anti-mouse or anti-rabbit IgGs conjugated to horseradish peroxidase (Amersham, UK) were incubated for 1 h at RT (1 : 10 000). Immunostained bands were detected by chemiluminescence method (Santa Cruz Biotechnology).

### Colony formation assay

Single-cell suspensions were plated in 35 mm plates (500 cells/plate for Pt45P1 andMiaPaCa-2; 700 cells/plate for HPDE and HPAF-II). After 1 day, cells were treated for 24 h with gemcitabine at the dose indicated. At the end of the incubation, the medium was replaced every 48 h. After 10 days, cells were fixed in methanol for 10 min, stained overnight with 5% Giemsa (Sigma-Aldrich), washed twice in PBS and dried. Pictures were taken using a digital camera to count and measure the colonies. Results represent the mean of at least three experiments±s.d.

### Polysomes-RNPs fractionation by sucrose gradients

Polysomes fractionation was performed as indicated previously.^21, 32^ Briefly, MiaPaCa-2 cells were homogenized in lysis buffer (100 mM NaCl, 10 mM MgCl_2_, 30 mM Tris-HCl [pH 7.5], 1 mM DTT, 30 U/ml RNasin) supplemented with 1% Triton X-100. After 5 min of incubation on ice, the lysates were centrifuged for 10 min at 12 000 × *g* at 4 °C. The supernatants (1.5 mg of protein extracts) were loaded on a 15–50% (wt/vol) sucrose gradients and sedimented by centrifugation for 110 min at 37 000 r.p.m. in a Beckman SW41 rotor (Fullerton, CA, USA). Each gradient was collected in 10 fractions, RNA was extracted by phenol/chloroform method. Fractions 1–5 (Polysomes) and 8–10 (RNPs) were pulled and analyzed by RT-PCR and qPCR methods.

### 3′ RACE PCR

Total RNA (1 *μ*g) was used for retrotranscription with 0.5 *μ*g of oligo oligo-dT tail followed by an anchor sequence.^26^ PolyA enriched/anchor tagged cDNA was subsequently used in RT-PCR experiments using gene-specific forward primers in presence of anchor reverse primer.

## Publisher's Note

Springer Nature remains neutral with regard to jurisdictional claims in published maps and institutional affiliations.
