# Supplementary material for: Alternative polyadenylation of ZEB1 promotes its translation during genotoxic stress in pancreatic cancer cells
Source: Cell Death Dis. 2017 Nov 9;8(11):e3168–. doi: 10.1038/cddis.2017.562 (PMC5775412; doi:10.1038/cddis.2017.562)
Supplement: Supplementary Information [file cddis2017562x1.pdf]

Alternative polyadenylation of ZEB1 promotes its translation during genotoxic stress in pancreatic cancer cells

Ilaria Passacantilli[1,2], Valentina Panzeri[1,2], Pamela Bielli[2,3], Donatella Farini[1], Emanuela Pilozzi[2], Gianfranco Delle Fave[2], Gabriele Capurso[2], and Claudio Sette[2,3].

[1]*Department of Biomedicine and Prevention, Section of Anatomy, University of Rome "Tor Vergata", Rome, Italy;*

[2]*Department of science medical/chirurgic and translational medicine, University of Rome "Sapienza", Rome, Italy;*

[3]*Laboratory of Neuroembryology, Fondazione Santa Lucia IRCCS, Rome, Italy.*

SUPPLEMENTARY INFORMATION

Table S1. List of oligonucleotides used in this study.

#	**Name**	**Oligo 5' -->3'**	
1	Anchor rv	CTGATCTAGAGGTACCGGATCC	3’RACE
2	Primer anchor	CTGATCTAGAGGTACCGGATCCTTTTTTTTTTTTTTTTTT	3’RACE
3	MYCN/5’UTR/EcoRI/FW	AAGAATTCGTCTGGACGCGCTGGGTGGATGCGGG	Cloning
4	N-MYC/5’UTR/NcoI/RV	TTTCCATGGTGGACGTGGAGCAGC	Cloning
5	E-CADHERIN FW	AGTTTTCCACCAAAGTCACGC	RT-PCR
6	E-CADHERIN RV	AGGAGTTGGGAAATGTGAGCA	RT-PCR
7	GAPDH FW	CCCTTCATTGACCTCAACTACATG	RT- and q-PCR
8	GAPDH RV	TGGGATTTCCATTGATGACAAGC	RT- and q-PCR
9	HPRT FW	TGACCAGTCAACAGGGGACA	RT-PCR
10	HPRT RT FW	TGCTGGATTACATCAAAGCACTG	qPCR
11	HPRT RT RV	TCCACCAATTACTTTTATGTCCCCT	qPCR
12	HPRT RV	TTCGTGGGGTCCTTTTCACC	RT-PCR
13	MYC FW	GCTTCTCTGAAAGGCTCTCCT	RT- and q-PCR
14	MYC REV	CACCGAGTCGTAGTCGAGGT	RT- and q-PCR
15	SLUG FW	AGTCCAAGCTTTCAGACCCCCATGCCATTG	RT-PCR
16	SLUG RV	TTCTCCCCCGTGTGAGTTCTA	RT-PCR
17	SLUG RT EX2 FW	CAAGGCGTTTTCCAGACCCTG	qPCR
18	SLUG RT EX3 RV	TTGACCTGTCTGCAAATGCTCT	qPCR
19	SNAIL FW	CACTATGCCGCGCTCTTTC	RT- and q-PCR
20	SNAIL RV	GCTGGAAGGTAAACTCTGGATTAGA	RT- and q-PCR
21	VIMENTIN FW	AGACACTATTGGCCGCCTGCAGGATG	RT-PCR
22	VIMENTIN RV	GAAGAGGCAGAGAAATCCTGCTCTCCTCGCCTTCCA	RT-PCR
23	ZEB1 Cost ex7 FW	ACTCAACTACGGTCAGCCCT	qPCR
24	ZEB1 Cost ex8 RV	TGGGCGGTGTAGAATCAGAG	qPCR
25	ZEB1 EX1C Cln FW	AGGAATTC TTTCTCCCTCCCCTCTGGGATG	Cloning
26	ZEB1 EX1C Cln RV	AGGAATTC AAAGCCACATCAGCAACAGCGGC	Cloning
27	ZEB1 exon 1 RT FW	CGAGCATTTAGACACAAGCGAG	qPCR
28	ZEB1 exon 1 RT RV	GTTATTGCGCCGCGGGTTC	qPCR

29	ZEB1 exon 1C RT FW	GCTGTTTCAAGATGTTTCCTTCCA	qPCR
30	ZEB1 exon 1C RT RV	ACAGACGTCTTTAAAATGCAAGTGT	qPCR
31	ZEB1 FW	CATTGCTGACCAGAACAGTGTTCC	RT-PCR
32	ZEB1 P1 fw	CTGATGAAGGATGACAGGGCT	qPCR
33	ZEB1 P1 rv	TCAGACACTTGCTCACTACTCTC	qPCR
34	ZEB1 P2 fw	ACATTTTGTGCCAATTTGTTCCTG	qPCR
35	ZEB1 P2 rv	TGACCATGATGTAACAAGGAACTT	qPCR
36	ZEB1 P3 fw	CCCCACTAGGAACAGGAACC	RT-PCR
37	ZEB1 P3 rv	CAACTTATGCCAGGCACCCT	RT-PCR
38	ZEB1 RV	TGGGCGGTGTAGAATCAGAGTCAT	RT-PCR
39	ZEB1 FW2	GAACCATCTTCTCCTGAACCAGGC	RT-PCR

Supplementary Figure 1. Gemcitabine treatment enhances ZEB1 expression in mesenchymal PDAC cell lines. A) Conventional RT-PCR analysis of E-cadherin and Vimentin expression in the indicated cell lines (HPDE, HPAF-II, Pt45P1, MiaPaCa-2). HPRT was used as loading control. B) Western blot analysis of E-cadherin and Vimentin expression in four different PDAC cell lines (HPDE, HPAF-II, Pt45P1, MiaPaCa-2). Coomassie staining was used as loading control. C) Dosage-response analysis to gemcitabine treatment preformed by colony formation assay (0,01 μM, 0,03 μM, 0,1 μM, 0,3 μM, 1 μM) in PDAC cell lines. Histograms represent the percentage of inhibition of colony formation in comparison to control cells. Statistical analysis was performed by ANOVA *** $P \leq 0.001$. D) Western Blot analysis of the pro-apoptotic cleavage of protein PARP-1. Actin was used as loading control.

Supplementary Figure 2. Structured 5'UTR of ZEB1 does not display IRES activity. A) Predicted structure of the Exon 1C-encoded 5'UTR sequence (392 bp) of ZEB1 by using The Mfold Web Service (http://unafold.rna.albany.edu/?q=mfold). B) Luciferase assay to monitor the translational activity of Exon 1C 5'UTR or MYC IRES sequences. The ZEB1 5'UTR 1C and c-MYC IRES sequences were cloned upstream to the Firefly gene reporter into the pRF plasmid and the constructs were transfected into MiaPaCa-2 Histograms represent the ratio between the Firefly and Luciferase in cells untreated of treated with gemcitabine 10 μM for 48 hours. Statistical analysis was performed by ANOVA *** $P \leq 0.001$. C) qRT-PCR analysis of the polysomal loading of ZEB1 transcripts containing Exon1C in MiaPaca-2 cells untreated of treated with gemcitabine 10 μM for 48 hours. Statistical analysis was performed by T-test student * $P \leq 0,05$.

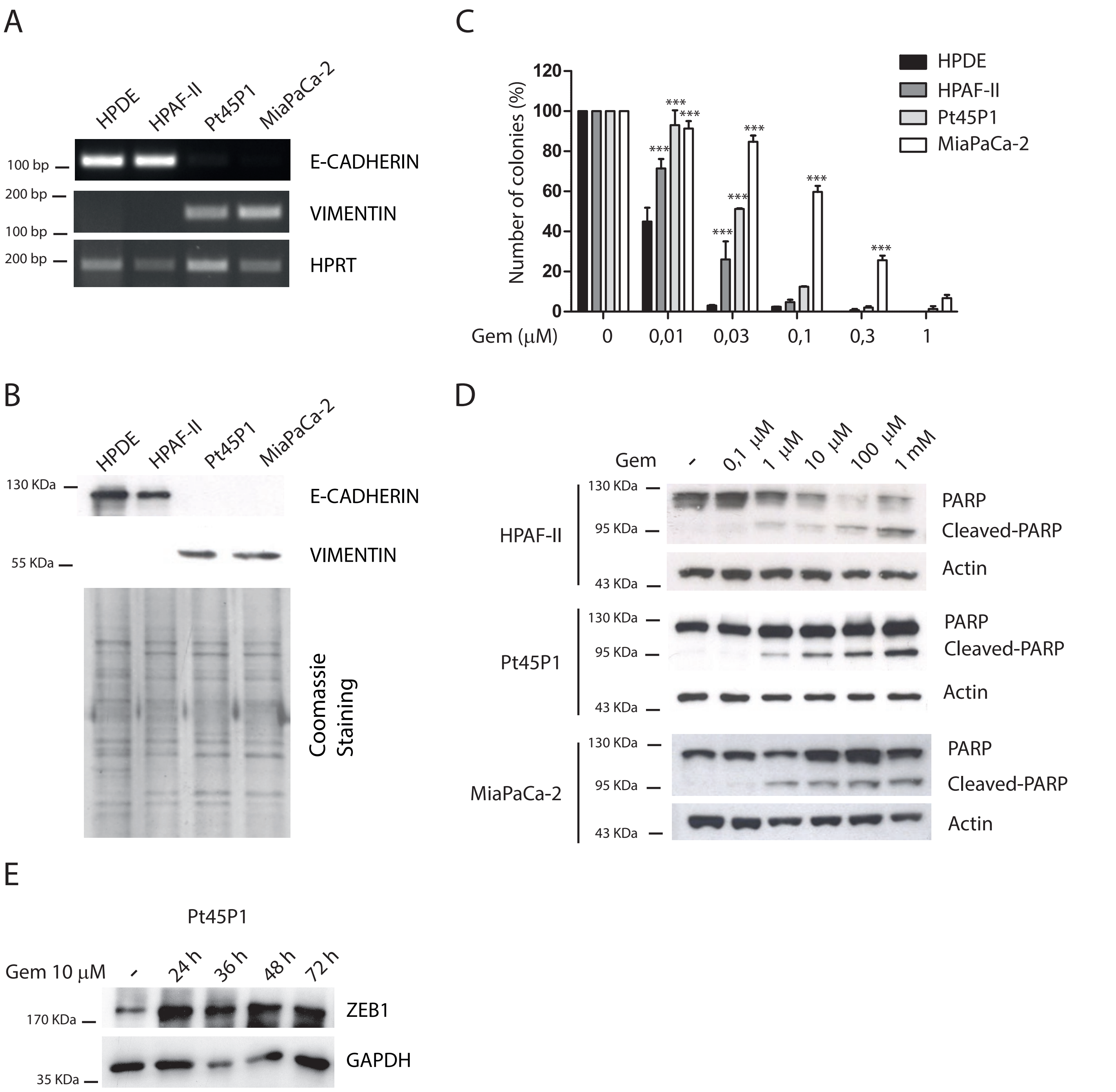

A
HPDE
HPAF-II
Pt45P1
MiaPaCa-2
100 bp
200 bp
100 bp
200 bp
E-CADHERIN
VIMENTIN
HPRT
B
130 KDa
55 KDa
Coomassie Staining
C
Number of colonies (%)
Gem (μM)
0
0,01
0,03
0,1
0,3
1
D
Gem
-
0,1 μM
1 μM
10 μM
100 μM
1 mM
HPAF-II
Pt45P1
MiaPaCa-2
130 KDa
95 KDa
43 KDa
PARP
Cleaved-PARP
Actin
E
Pt45P1
Gem 10 μM
24 h
36 h
48 h
72 h
170 KDa
35 KDa
ZEB1
GAPDH

Figure S1

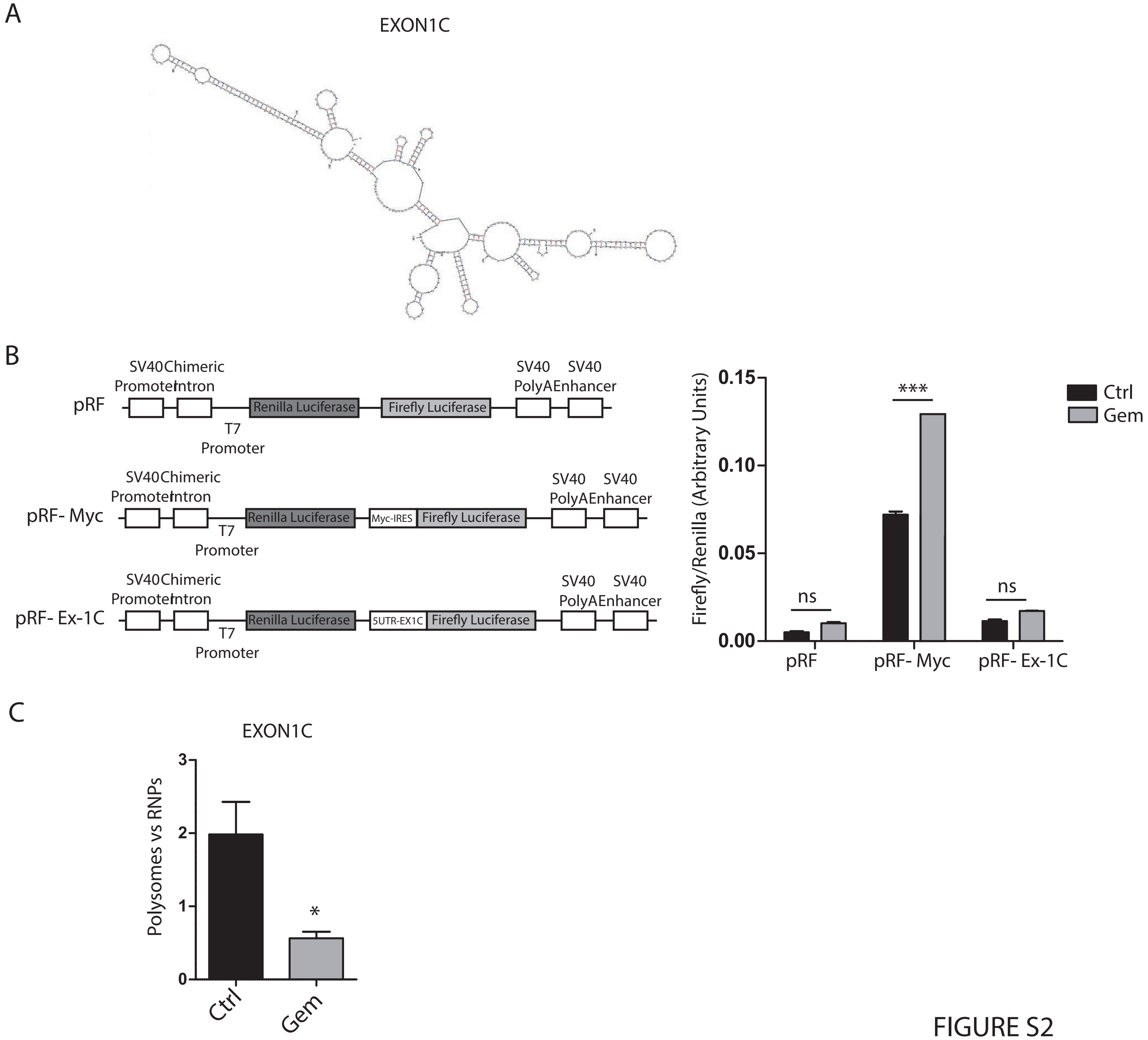
A
EXON1C
B
SV40 Promoter
Chimeric Intron
pRF
Renilla Luciferase
Firefly Luciferase
SV40 PolyA
SV40 Enhancer
T7 Promoter
pRF- Myc
Myc-IRES
pRF- Ex-1C
5UTR-EX1C
Firefly/Renilla (Arbitrary Units)
0.15
0.10
0.05
0.00
ns
***
Ctrl
Gem
C
EXON1C
Polysomes vs RNPs
3
2
1
0
*

FIGURE S2
